# A Study of Human Papillomavirus Genotypes Specificity and the Vaccines Introducing System for Korean-Chinese Women in the Yanbian Autonomous Region

**DOI:** 10.31557/APJCP.2020.21.11.3331

**Published:** 2020-11

**Authors:** Qunying Wu, Jin-Ho Kuk, Young-Joon Ryu

**Affiliations:** 1 *Department of Pathology, College of Medicine, Kangwon National University, Chun-Cheon, Republic of Korea. *; 2 *Department of Pathology, Yanbian Maternal and Child Health Care Hospital, Yanji, China. *

**Keywords:** Human papillomavirus, genotype, vaccine, law

## Abstract

**Objectives:**

Human papillomavirus (HPV) is a major cause of cervical cancer in women. The characteristics of HPV infection vary; therefore, it is necessary to identify the most common HPV genotypes among a group of subjects when introducing a vaccine program. Currently, in the Yanbian Autonomous Region, no HPV vaccinations are not provided, and no data has been reported regarding HPV rates or genotype prevalence. We aimed to find the most suitable HPV vaccine for this region and reasons why no vaccine has been introduced.

**Methods:**

HPV genotyping of 200 Korean-Chinese women living in the Yanbian Autonomous Region who visited the hospital for annual health examination was done. We also checked main factors necessary for HPV vaccine administrative system; (1) vaccine manufacturers in China, (2) vaccine importers, (3) vaccine suppliers, (4) applicable vaccine laws, (5) the HPV vaccine permit system in Jilin Province, and (6) vaccination hospital facilities-were assessed by direct inquiry and search.

**Results:**

The results showed that HPV genotypes 52, 58, 16, 53, and 33 were the most common among Korean-Chinese women. These results differed from those previously reported for Korean or Chinese women. All elements necessary for introduction of HPV vaccine were prepared, but there is no HPV vaccination plan based on epidemiological investigation.

**Conclusions:**

Gardasil® 9 should be the most suitable vaccine for Korean-Chinese women with HPV infection and cervical cancer in this region considering the prevalence of certain genotypes. Governments and medical institutions should take an active stance on HPV vaccination to lower the incidence of cervical cancer here. Our study may serve as an important reference for introducing a Chinese government program designed to prevent cervical cancer.

## Introduction

Cervical cancer is one of the leading causes of death for women and occurs frequently around the world (Torre et al., 2015). Human papillomavirus (HPV) has been identified as a major cause of squamous cell cervical cancer or precursor lesions and is observed in 90 % of cervical cancer patients (Walboomers et al., 1999). There are more than 200 HPV genotypes, 40 of which cause infection of the reproductive system and some of which are known to be carcinogenic (de Villiers EM et al., 2004; Munoz at al., 2003; Hutchinson at al., 2008; Markowitz et al., 2014; Silviade et al., 2018). Depending on how each genotype impacts the progression towards a malignant tumor, HPV genotypes are characterized as either high-risk or low-risk (Wells et al., 2003; Markowitz et al., 2014; Silviade et al., 2018). Continued infection with high-risk HPV increases the risk of potential intraepithelial lesions, even though there may be no histological change in the cervix (Rozendaal ea al., 1996; Ylitalo et al., 2000; Woodman et al. 2001; Castle et al., 2002; Naucler et al., 2007; Schiffman et al., 2009). The incidence of cervical cancer in China has continued to rise by 11.3 % annually on average for the past several years. According to the 2015 Chinese Cancer Registry Annual Report, 98,900 people develop cervical cancer each year, with 30,500 deaths (Chen et al., 2016). While many studies have reported HPV infection data for China as a whole, no study of the HPV positive rates and genotypes has been conducted on Korean-Chinese women living in the Yanbian Autonomous Region.

Some vaccines have been developed and marketed around the world to prevent common HPV genotypes that potentially cause cervical cancer. These vaccines include Cervarix® 2 (GlaxoSmithKline (GSK) Biologicals) and Gardasil® 4 and 9 (Merck Sharp & Dohme). Each vaccine can prevent only particular HPV genotypes (Kim et al., 2009; Petrosky et al., 2015). Cervarix^®^2 is effective for HPV genotypes 16 and 18, Gardasil^®^4 is effective for HPV genotypes 6, 11, 16, and 18, and Gardasil^®^9 is effective for HPV genotypes 6, 11, 16, 18, 31, 33, 45, 52, and 58 (Kim et al., 2009; Doorbar et al., 2012; Petrosky et al., 2015; Drolet et al., 2015). Although HPV vaccines are very effective in preventing cervical cancer, they are not yet sufficiently available in different parts of the world. While an HPV vaccination program began in Hong Kong in 2006 for the first time in China (Tay et al., 2008; Li et al., 2013), it was not until after 2016 that the vaccines were approved by the China Food and Drug Administration (CFDA) and introduced in mainland China. Cervarix^®^2 was officially approved for introduction in July 2016 (Ma et al., 2019), Gardasil^®^4 in May 2017, and Gardasil^®^9 in April 2018 (Liu and Chui, 2018; Ma et al., 2019). Nonetheless, except for in some health facilities in large cities like Beijing, it was found that vaccination is not provided in most cities across China, and no HPV vaccine has been introduced in the Yanbian Autonomous Region. 

HPV infection is affected by geographical location, race, immunological condition, and genetic factors (de Freitas et al., 2012; Laudadio, 2013; Zhang et al., 2016; Senapati et al., 2017). In this regard, we hypothesized that Korean-Chinese women may be affected by HPV differently than Korean and Chinese women, even though they are similar to Korean women in terms of race and live in the same region as Chinese women. If vaccination is to be made available for Korean-Chinese women living in the Yanbian Autonomous Region, knowing their HPV prevalence or genotypes would be important for selecting the most effective vaccine. The purpose of this study was to establish a basic reference for introducing HPV vaccination, which is essential for preventing cervical cancer, by investigating the HPV prevalence and genotypes of Korean-Chinese women in the Yanbian Autonomous Region, Jilin Province, China, and reviewing the administrative data required to introduce the vaccine. 

## Materials and Methods

*Patients*

This experiment was conducted on 200 Korean-Chinese female patients living in the Yanbian Autonomous Region who visited the Department of Obstetrics and Gynecology Yanbian Maternity and Child Health Care Hospital in Jilin Province for a Pap smear test from April 2018 to October 2018. All 200 Korean-Chinese women are living in the Yanbian Autonomous Region and had no previous gynecological history. They visited the hospital for annual health examination and underwent cervical cytology examination. This study was conducted with approval from the Institutional Review Board of Kangwon National University.

*HPV genotype test*

The samples collected from all the 200 subjects were subjected to cervical fluid cytology E-Prep 1000 (Jilin Shiye Medical Technology Co. Ltd., China) and HPV DNA Test (Hybribio Limited, Chauzou, China) at the same time. Tissue sampling and slide preparation was performed according to the protocol provided with the E-Prep 1000 Test. The prepared slides were first assessed by a pathologist and then reassessed by another pathologist. All abnormal readings were reassessed, and the diagnosis was confirmed by three pathologists working together. Cytological diagnosis was performed using the 2014 Bethesda System and samples were categorized as follows: low-grade squamous intraepithelial lesion (LSIL), high-grade squamous intraepithelial lesion (HSIL), atypical squamous cells of undetermined significance (ASCUS), atypical squamous cells, cannot exclude HSIL (ASC-H), squamous cell carcinoma (SCC), or adenocarcinoma (AC).

HPV genotyping was performed using a 23 HPV Genotyping Real-Time PCR Kit (Hybribio, HBRT-23, Hong Kong). This test can detect 15 high-risk HPVs (16, 18, 31, 33, 35, 39, 45, 51, 52, 53, 56, 58, 59, 66, 68) and 8 low-risk HPVs (6, 11, 42, 43, 44, 81, 73, 82), allowing analysis of a total of 23 HPV genotypes. After processing the sample, the extracted DNA was combined with real-time PCR master mix containing the primers provided in the kit, and gene amplification was performed according to the protocol provided by the manufacturer. If the Ct value for globulin, which was used as an internal control, was below 40 cycles, and the Ct value of the other sequences was not clear, the result was interpreted as negative. When the Ct value of the sample was 40–45 cycles, the sample was evaluated again. If the Ct value in the second test was less than 40, the sample was considered to be positive for HPV, otherwise, it was interpreted as negative. If the Ct values of were not checked, the results were considered invalid and the HPV genotype test of the sample used for examination of the cervical cytology was performed again. 

*Selection of the HPV vaccine*

Of the genotypes preventable by the three currently marketed HPV vaccines, those most frequently observed among the results of the HPV genotype test were used to choose the relevant vaccine.

*Evaluation of the main factors required for introduction of a vaccine*

**Figure 1:**
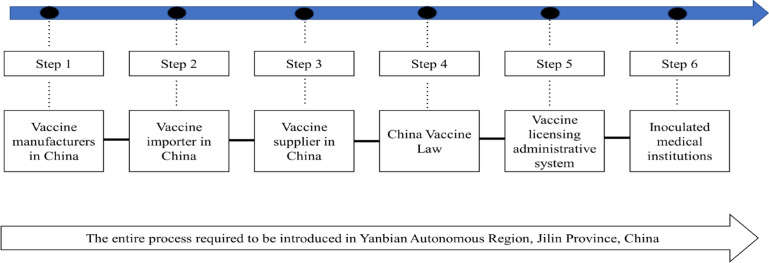
Administrative Steps for Vaccine Introduction

To find out why no vaccine has been introduced to the Yanbian Autonomous Region, we studied the process of vaccine introduction, including vaccine manufacturing, vaccine supply, vaccination permit authority, applicable vaccine law, conditions for vaccination facilities, first aid for accidents, compensation rules, and supervision rules (Figure 1). To gather information about each component of the process, we searched Chinese government agencies online, conducted a phone call questionnaire survey, and carried out an interview with relevant stakeholders. To identify the relevant government authorities, we examined the Ministry of Justice of the People’s Republic of China, China Legal Information Center, Ministry of Civil Affairs of the People’s Republic of China, National Health Commission of the People’s Republic of China, Yanbian Autonomous Region People’s Government, and Yanji People’s Government. Furthermore, we obtained information from government data that was available publically. Although we searched for vaccine manufacturers and suppliers online using various combinations of keywords (HPV, vaccine, Gardasil, Cervarix, and China) on PubMed, Web of Science, IMS, Scopus, and Google Scholar, very little information could be obtained. This was because of non-disclosure of information on the Internet by the Chinese government. Therefore, we collected data from relevant persons through phone calls or personal interviews.

## Results

*Patient demographic characteristics, HPV-positive rates, and HPV genotype test results*

Among the 200 patients, the mean age was 45.7 years, and the HPV-positive rate by age group was the highest for those aged 30-39 years, at 10.0 %, followed by 8.0% for those aged 60 or older, 7.0 % for those aged 40-49, 6.5 % for those aged 50-59, and 3.0 % for those aged 20-29. The total HPV-positive rate was 34.5 % (69/200), and the most common high-risk HPV genotypes, in descending order, were genotypes 52, 58, 16, 53, and 33 (Table 1). 

*Comparison of HPV-positive rates and genotypes among different regions across China and Korea*

The total HPV-positive rate (34.5 %) for Korean-Chinese women living in the Yanbian Autonomous Region was higher than the high-risk HPV-positive rate (21.07 %) in 37 cities across China (Wang et al., 2015). It was lower than the rates in Gangwon, Korea (72.2 %) (Lee et al., 2011) and Seoul (40.6 %) (So et al., 2016) but higher than the rates in 13 cities across Korea (27.8 %) (Nah et al., 2017) and in Daejeon and Daegu (26.6 %) (Hong et al., 2009) (Table 2).

The results of our HPV genotype test showed that the most frequent HPV genotypes were genotypes 52, 58, 16, 53, and 33, unlike those reported in Gangwon, Korea (16, 53, 58, 56, and 70) (Lee et al., 2011), Seoul (genotypes 53, 52, 58, 16, and 68) (So et al., 2016), 13 cities across Korea (genotypes 52, 58, 16, 56, and 51) (Nah et al., 2017), and Daejeon and Daegu (genotypes 16, 58, 18, 52, and 56 (53)) (Hong et al., 2009). Furthermore, the genotypes observed in our sample were different from those reported in 37 cities across China (genotypes 16, 52, 58, 59, and 39) (Wang et al., 2015) (Table 3).

*Selection of the HPV vaccine*

Among the other currently marketed vaccines (Table 4), Gardasil^®^9 is the only suitable vaccine for genotypes 52, 58, 16, 53, and 33, the most frequently observed genotypes in our sample. Therefore, we selected Gardasil^®^9 for further testing.

*Status of HPV vaccine manufacturers, suppliers, and permit authority in China*

**Figure 2:**
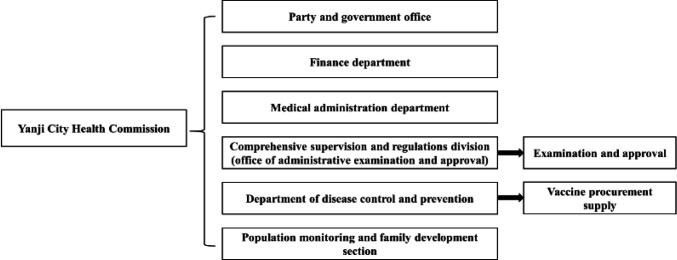
Institutional Map of Yanji City Health Commission

We found that while there are international vaccine manufacturers in China such as Merck Sharp & Dohme (MSD) Corp from New Jersey, USA, and GlaxoSmithKline Biologicals SA from Rixensart, Belgium, currently no vaccine manufacturer produces MSD’s Gardasil^®^9 in China. We confirmed that as a vaccine importer, Chongqing Zhifei Biological Products (300122.SZ) signed a supplementary agreement with MSD on May 1, 2018, to import, sell, and distribute Gardasil^®^9 across mainland China (Zhang, 2018). The following describes the study results for components required to introduce a vaccine, such as the vaccination permit authority, applicable vaccine law, conditions for vaccination facilities, first aid for any accident, compensation rules, and supervision rules: 

To introduce an HPV vaccine in the Yanbian Autonomous Region, a permit is required from the Comprehensive Supervision and Regulations Department of the Yanji City Health Commission, and vaccine procurement is subject to supply supervision by the Department of Disease Control and Prevention of the Yanji City Health Commission (Yanji People’s Government, 2019; Yanji center for disease control and prevention, 2008). The department responsible for health administration across China is the National Health Commission under the State Council of the People’s Republic of China, a central government agency. The Center for Disease Control and Prevention under the National Health Commission connects different regional provinces, prepares a serious disease prevention and treatment plan and a national immunization plan, organizes and implements interventions for problems that pose grave harm to people’s health and public sanitation, supplements the disease control and prevention system, and announces information about infectious disease breakouts (Central People’s Government of the People’s Republic of China State Council, 2018) (Figure 2). 

*Vaccine Management Law of the People’s Republic of China*

**Figure 3:**
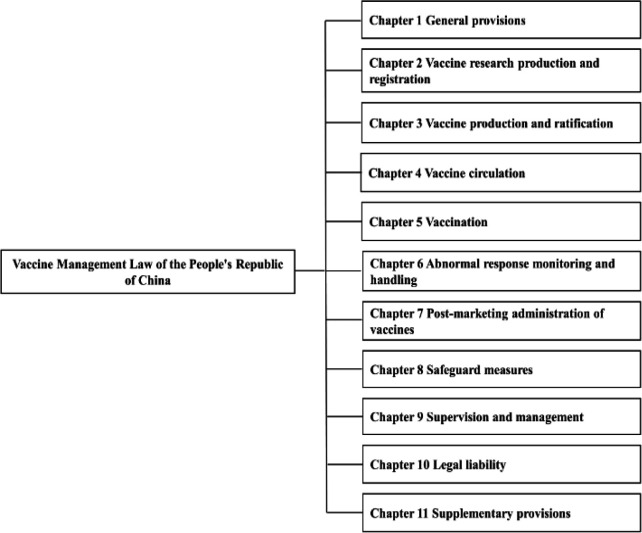
Contents List of (Vaccine Management Law of the People’s Republic of China)

The qualification and operation of vaccination facilities are defined in the Vaccine Management Law of the People’s Republic of China (Ministry of Justice of the People’s Republic of China, 2019). This law consists of General Provisions, Vaccine Research Production and Registration, Vaccine Production and Ratification, Vaccine Circulation, Vaccination, Adverse Reaction Monitoring and Handling, Post-Marketing Administration of Vaccines, Safeguard Measures, Supervision and Management, Legal Liability, and Supplementary Provisions (Fig. 3). Under this law, a “vaccine” refers to a preventative biological product used to immunize the human body to control and prevent the occurrence and spread of disease pursuant to Article 2, which includes an immunization plan vaccine and a non-immunization plan vaccine. 

As for vaccine procurement under Article 32, the Public Health Department of the State Council hosts competitive bidding or makes a collective deal for national immunization plan vaccines along with the Financial Department of the State Council, and each province, autonomous region, and city procures the vaccines in a uniform way. Each provincial and local government must examine and report vaccine demand, which is defined in Article 34. Vaccine supply is set forth in Article 35 of Chapter 4, while vaccine shipment and cost are defined in Article 36. The Financial Department and Price Department of the State Council determine the specific method for calculating cost, while the price departments of the provincial, autonomous region, and city governments establish the criteria for collecting money to pay for the cost along with the Financial Department. 

The permit for vaccination facilities is specified in Article 44: (1) A healthcare facility business permit must be acquired, (2) the facility must have a physician, nurse, or village doctor professionally trained and certified by the Public Health Department in the prefectural-level People’s Government, and (3) the facility must be equipped with refrigeration facilities, equipment, and systems in compliance with vaccine storage, transport, and management requirements. The Public Health Department of the People’s Government above the prefectural level designates a healthcare facility that satisfies the requirements to perform an immunization plan vaccine program in its jurisdiction. Healthcare facilities that satisfy the requirements are allowed to perform a non-immunization plan vaccine program but must report to and register with the Public Health Department, which issues a business permit to the facility. The vaccination unit must strengthen internal control and comply with a vaccination work code, procedures, guidelines, and practices when implementing a vaccination program, and the disease control and prevention body at each level must provide technical guidance and vaccine control for the vaccination unit’s vaccination program. 

Currently, under the Vaccine Management Law, candidate healthcare facilities that can provide vaccination in the Yanbian Autonomous Region include Yanbian University Hospital and the Yanbian Maternity and Child Health Care Hospital affiliated with Yanbian University Hospital (Table 3).

**Table 1:** Difference in Human Papillomavirus Prevalence between Korean-Chinese Women and Korean Women

HPV Genotyping Result	HPV prevalence No(%)				
	Korean-Chinese women	Gangwon Korean women	South Korean women	Women from 13 cities in Korea	Korean women from Daejeon, Daegu, etc
HPV positive No (%)	69 (34.5)	247 (72.2)	393 (40.6)	5227 (27.8)	629 (26.6)
HPV negative No (%)	131 (65.5)	95 (27.8)	575 (59.4)	13588 (72.2)	1739 (73.4)
Total No (%)	200 (100)	342 (100)	968 (100)	18815 (100)	2368 (100)

No, Number of patients

**Table 2:** Coverage by Different Vaccines of Human Papillomavirus Genomic Subtypes in Korean-Chinese Women

	Cervarix 2	Gardasil 4	Gardasil 9
Manufacturer	Glaxo Smith Kline	Merck	Merck
Contains VLP form	16,18	6,11,16,18	6,11,16,18,31,33,45,52,58
Coverable HPV subtypes common to Korean-Chinese women	16	16	52,58,16,33

VLP, Virus-like particles

**Table 3:** Six Necessary Check Points in the Human Papillomavirus Vaccine Introduction Process

Required factors		Factors present in China
Vaccine manufacturers	Authorized Company	None
Vaccine importer	Authorized Company	Merck Sharp & Dohme and GlaxoSmithKline Biologicals
Vaccine supplier	Authorized Company	Chongqing Zhifei, China. 300122.SZ
Vaccine permit system	Government	Vaccine Management Law of the People's Republic of China
Authorized government office organization	Government	Health Commission
Enforced medical institution designation system	Law	Vaccine Management Law of the People's Republic of China in accordance with Chapter 5, Article 44, the agency must apply.
Vaccination Enforcement Law	Provision	Current Situation in Hospital
	Vaccination Object Range	Only pediatric vaccinations
	Space for Injection	Only for newborns. No adult
	Facility for Storage etc.	Only for newborns. No adult
	Professional Manpower	Only for newborns. No adult
	Emergency Treatment Capacity	Available
	Compensation Regulations for Accidents	Available
	Management Supervision System	Present

**Figure 4:**
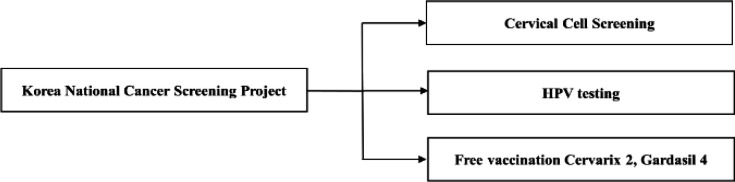
Korea National Cancer Screening Project

## Discussion

According to this study’s results, the HPV-positive rate among Korean-Chinese women in the Yanbian Autonomous Region, Jilin Province, China was higher than the high-risk HPV-positive rate in 37 cities across China, lower than the HPV-positive rate in Gangwon and Seoul, Korea, and slightly higher than the HPV-positive rate in 13 cities across Korea as well as in Daegu and Daejeon. In addition, the five most common HPV genotypes among Korean-Chinese women were genotypes 52, 58, 16, 53, and 33, which were different those reported in Chinese women in 37 cities across China, in Seoul, in Gangwon, in Daejeon and Daegu, and in 13 cities across Korea. These results were consistent with the previous hypothesis that epidemiological diversity in HPV infection rates and genotypes varies depending on country, region, race, genetic factors, and immunological conditions (de Freitas et al., 2012; Laudadio, 2013; Zhang et al., 2016; Senapati et ak., 2017), and therefore, indicated that a vaccine to prevent HPV among Korean-Chinese women living in Yanbian Autonomous Region should be selected based on factors unique to the region. In this study, the most frequently observed HPV genotypes among Korean-Chinese women were 52, 58, 16, 53, and 33. Gardasil^®^9 targets HPV genotypes 6, 11, 16, 18, 31, 33, 45, 52, and 58, and we found that only this vaccine was expected to be effective in preventing cervical lesions in Korean-Chinese women; since Cervarix^®^2 targets HPV genotypes 16 and 18 and Gardasil^®^4 targets HPV genotypes 16, 18, 6, and 11, they would only target one of the relevant genotypes 16. 

In Korea, the National Cancer Prevention Program provides free vaccination with Gardasil® 4, but Gardasil^®^9 is provided selectively at a cost to the individual. Despite the high price of Gardasil®9, other HPV vaccines are not currently expected to be effective for Korean-Chinese women. A post-clinical trial follow-up study of 1,717 Asian patients (307 Korean patients) for 4.5 years evaluated the efficacy and safety of Gardasil®9. In the Gardasil®9 treatment group, 0 lesions were reported in the cervix, vulva, or vagina that were related to HPV genotypes 31, 33, 45, 52, and 58, and 0 cases of continued infection were reported (Korea Pharmaceutical Bio, 2019; Kim et al., 2019). In a clinical study, Gardasil®9 was shown to be highly effective, with an efficacy of 97.4 % for preventing cervical, vaginal, and vulvar diseases caused by HPV genotypes 31, 33, 45, 52, and 58 (Joura et al., 2015).

The present study also examined the components of the process required to introduce the HPV vaccine in the Yanbian Autonomous Region and confirmed that while there is no production facility for Gardasil^®^9 in China, there is an importer that imports the vaccine and a supplier that safely delivers it. The applicable law took effect in 2019, at which time the relevant authority began to function. In fact, Gardasil^®^9 was provided at United Family Healthcare in Boao, Hainan Province, on May 30, 2018, for the first time in China outside of Hong Kong. It is not confirmed whether the vaccine can be provided widely in practice, although the vaccine was made available in Beijing, Shanghai, Guangzhou, Shenzhen, Hangzhou, and Chongqing and ratification was issued retrospectively (Chinese Mom Help Community, 2018; Nie, 2018; Feng, 2020). Until now, however, vaccination has not been performed in parts of the country other than in large cities like Beijing, and no vaccine has been introduced in the Yanbian Autonomous Region. To confirm these findings, we requested specific data such as the number of vaccinations, but vaccination providers do not reveal any information, nor does the government publish an official report. Therefore, it remains unclear why Gardasil^®^9 is not actually provided in practice, despite the supply permit granted in 2018. 

We also examined the National Cervical Cancer Screening Program implemented by the Korean government and found that the National Cancer Center formed the Cervical Cancer Screening Recommendations Amendment Committee in 2013 to provide recommendations from academia, develop evidence-based screening recommendations for cervical cancer, provide standard cervical cancer screening guidelines to healthcare professionals, and amend cervical cancer screening recommendations for 3 years with the goal of providing people with suitable information about the benefit and risk of cervical cancer screening. The Cervical Cancer Screening Program, which traditionally provided a Pap smear test to women of age 30 years or older every 2 years, expanded the age group to include women of age 20 years or older based on newly developed evidence-based screening recommendations made in January 1, 2016 (Kim et al., 2019). Korean women of age 20 years or older can now receive a Pap smear test for free once every 2 years. According to Article 2 of the Infectious Disease Control and Prevention Act in Korea (National Legal Information Center, 2020), HPV infection is classified as “Class 4 infectious disease” and was included in the scope of national vaccination in 2016, with Cervarix^®^2 and Gardasil^®^4 currently provided for free as part of the Healthy Women First Step Clinic Program (Figure 4). Article 24 of Chapter 6: Vaccination states that the Special Self-Governing Province Governor or the head of a Si/Gun/Gu shall provide mandatory vaccination at public health clinics under his/her jurisdiction and may entrust a medical institution within his/her jurisdiction with the affairs of mandatory vaccination as prescribed by a presidential decree. In Korea, Gardasil^®^9 obtained marketing authorization from the Ministry of Food and Drug Safety in January 2016, accounted for a market share of 6 % right after its launch, and increased its market share up to 40 % in 2018. Some argued that Gardasil^® ^9, which was developed after Gardasil^®^, be included in nationally required vaccinations (Jang, 2018). The principles of the Cervical Cancer Screening Program in Korea may be applicable to preventing cervical cancer in Korean-Chinese women in the Yanbian Autonomous Region; it would be helpful to develop a government-led cancer prevention program in this region that facilitate more vaccinations in addition to the Pap smear and HPV gene tests currently available in the private sector. 
